# Diagnostic Accuracy of Serum Cystatin C for Early Recognition of Nephropathy in Type 2 Diabetes Mellitus

**DOI:** 10.1155/2021/8884126

**Published:** 2021-04-26

**Authors:** Suman Sapkota, Saroj Khatiwada, Shrijana Shrestha, Nirmal Baral, Robin Maskey, Shankar Majhi, Lal Chandra, Madhab Lamsal

**Affiliations:** ^1^Lumbini Zonal Hospital, Butwal, Nepal; ^2^School of Medical Sciences, UNSW Sydney, Kensington, Australia; ^3^Department of Biochemistry, B P Koirala Institute of Health Sciences, Ghopa, Dharan, Nepal; ^4^Department of Internal Medicine, B P Koirala Institute of Health Sciences, Dharan, Nepal; ^5^Department of Biochemistry, Maulana Azad Medical College, New Delhi, India

## Abstract

**Objectives:**

Diabetic nephropathy is one of the major complications that develop over time in type 2 diabetes mellitus (T2DM). This prospective study was conducted to assess the diagnostic accuracy of serum cystatin C in detecting diabetic nephropathy at earlier stages.

**Materials and Methods:**

This study was undertaken on 50 cases of T2DM and 50 healthy subjects as controls. Demographic and anthropometric data and blood and urine samples were collected. The concentration of serum cystatin C (index test) and traditional markers of diabetic nephropathy, serum creatinine, and urinary microalbumin (the reference standard) were estimated. Similarly, blood glucose, glycated haemoglobin (HbA1c), triglycerides, total cholesterol, high-density lipoprotein (HDL) cholesterol, and urinary creatine were measured.

**Results:**

The mean ± SD serum cystatin C was significantly higher in T2DM as compared to control (1.07 ± 0.38 and 0.86 ± 0.12 mg/dl, respectively, *p* < 0.001). The mean ± SD bodyweight, BMI, W : H ratio, pulse, SBP, and DBP were 66.4 ± 12.6 kg, 26.2 ± 5.6 kg/m^2^, 1.03 ± 0.09, 78 ± 7, 125 ± 16 mm of Hg, and 77 ± 9 mm of Hg, respectively, in cases. A significant difference in HDL cholesterol (*p*=0.018) and serum cystatin C (*p* < 0.001) was observed among different grades of nephropathy. Cystatin C had a significant positive correlation with age (*r* = 0.323, *p*=0.022), duration of T2DM (*r* = 0.326, *p*=0.021), and UACR (*r* = 0.528, *p* < 0.001) and a significant negative correlation with eGFR CKD-EPI cystatin C (*r* = −0.925, *p* < 0.001). The area under ROC curve for serum cystatin C (0.611, 95% CI: 0.450–0.772) was greater than for serum creatinine (0.429, 95% CI: 0.265–0.593) though nonsignificant.

**Conclusion:**

Serum cystatin C concentration increases with the progression of nephropathy and duration of diabetes in Nepalese T2DM patients suggesting cystatin C as a potential marker of renal impairment in T2DM patients.

## 1. Introduction

Diabetes mellitus (DM) has reached an epidemic state; globally, 1 in 11 adults have DM (90% have type 2 diabetes mellitus (T2DM)) [1]. This has led to an increase in microvascular (nephropathy, retinopathy, and neuropathy) and macrovascular (coronary artery disease, peripheral arterial disease, and stroke) complications [2, 3]. Up to 40% of DM patients are said to be affected by diabetic nephropathy (DN), and the diagnosis is usually made based on the presence of albuminuria (increased urinary albumin excretion (UAE)) and/or reduced estimated glomerular filtration rate (eGFR) in the absence of other renal diseases [4–6].

Diabetic nephropathy is seen as one of the major complications in Nepalese DM patients, a study reported DN in 16.6% of patients [7]. In another study among T2DM cases, 14.7% had frank proteinuria, 44.64% had microalbuminuria, and the rest without proteinuria [8]. Commonly measured markers for the diagnosis and progression of DN include serum creatinine, eGFR, blood urea, and urinary albumin [9, 10]. Early detection of abnormal renal function is essential to slow down the progression to nephropathy stage and further to end-stage renal disease (ESRD). Many markers such as cystatin C, alpha 1-microglobulin, immunoglobulin G or M, angiotensinogen, liver-type fatty acid-binding protein, urinary transferrin, serum osteopontin, urinary retinol-binding protein, and interleukin-18 have been screened as early indicators of DN [6, 9–13].

Cystatin C is a prominent marker whose efficacy has been assessed for early diagnosis and progression of DN [14–20]. Despite emerging data on cystatin C as a promising DN marker in different populations, it has not been studied much for its usefulness and applicability in detecting DN and estimating eGFR in Nepalese community. Race/ethnicity is found to affect cystatin C levels in the normal population [21], and the prevalence of chronic kidney disease (CKD) estimated using cystatin C [22]. Some studies have suggested using cystatin C and creatinine combination equation for estimating GFR in multiethnic Asian patients with CKD to avoid the need to use ethnicity coefficient [23, 24].

A previous study in the Nepalese population tested cystatin C as a marker of renal impairment in preeclampsia [25]. Here, we plan to estimate cystatin C in a healthy population and T2DM patients. Specifically, we aimed to assess the diagnostic accuracy of cystatin C for diagnosing DN by comparing it with the traditional markers of nephropathy such as albuminuria, serum creatinine, and serum creatinine-based eGFR in the Nepalese population.

## 2. Materials and Methods

This cross-sectional study was conducted in a hospital-based setting of B P Koirala Institute of Health Sciences (BPKIHS), Dharan, Nepal. Fifty cases of T2DM and 50 healthy subjects as controls were recruited in this study. The cases were either newly identified T2DM patients based on the American Diabetes Association (ADA) criteria or patients already on T2DM medications [4]. The exclusion criteria were patients with urinary tract infection, hypothyroidism or hyperthyroidism, and pregnancy. Subjects for the control group were apparently healthy hospital staff and visitors and were nondiabetics. Written consents were taken from each subject before enrolling in the study. The ethical clearance for conducting this study was provided by the Institutional Review Committee of BPKIHS, Dharan, Nepal.

### 2.1. Data Collection

Subjects were recruited randomly, and while recruiting subjects, details were recorded. This included age, sex, dietary habit, alcohol and smoking behaviour, the family history of T2DM, duration of T2DM, the medication used (oral hypoglycaemic agents, insulin or both), and complications of T2DM (retinopathy, neuropathy, and nephropathy). The anthropometric variables (height, weight, waist circumference, and hip circumference) were measured using standard techniques, and body mass index (BMI) (weight in kg/height square in meters) was calculated. Pulse and blood pressure (BP) (systolic and diastolic BP) were measured using standard procedures.

### 2.2. Sample Collection

After the selection of cases and controls, blood and urine samples were collected. Blood samples were collected by venepuncture. Fasting blood glucose was estimated after 8 hours of fasting and postprandial blood glucose after 2 hours of meal. HbA1c was estimated from blood samples collected in K2EDTA vial. Other biochemical analytes such as cystatin C, creatinine, triglycerides, total cholesterol, and high-density lipoprotein (HDL) cholesterol were measured in the serum. For serum separation, blood was collected in plain vial and centrifuged at 3000 rpm for 10 minutes. Serum was aliquoted and stored at −20°C until biochemical analysis. Spot urine samples were collected for the determination of urinary creatinine and microalbumin. Samples were immediately centrifuged at 2000 rpm for 10 minutes, and the supernatant was used for biochemical analysis.

### 2.3. Laboratory Tests

Blood glucose, serum and urinary creatinine, and blood HbA1c were measured by the Cobas c311 Roche-Hitachi chemistry analyzer using Roche reagents. Serum cystatin C was measured by using the Accent-200 fully automated analyzer and urinary microalbumin by the Nycocard analyzer. Estimation of blood sugar, serum and urinary creatinine, HbA1c, cystatin C, and urinary microalbumin was performed by the hexokinase method, Jaffe reaction, turbidimetric inhibition immunoassay, immuno-turbidimetric, and solid-phase immunometric methods, respectively. After the estimation of these biochemical parameters, several ratios were calculated. Based on the albumin : creatinine ratio, patients were divided into three categories as those having normoalbuminuria (albumin : creatinine ratio <30 mg/g), microalbuminuria (albumin : creatinine ratio 30–300 mg/g), or macroalbuminuria (albumin : creatinine ratio >300 mg/g) [5].

### 2.4. eGFR Calculation

Based on the serum creatinine values, eGFR was calculated using several equations described as follows. Cockcroft-Gault (CG) equation used formula eGFR = (140 − age) × weight (kg) / (72 × SCr (mg/dL)) × (0.85 if female) [26]. Serum creatinine-based chronic kidney disease epidemiology collaboration (CKD-EPI) used formula eGFR = 144 × (SCr/0.7)^−0.329 (−1.209 if SCr > 7)^ × 0.993^age^ for females and eGFR = 141 × (SCr/0.9)^−0.411 (−1.209 if SCr > 0.9)^ × 0.993^age^ for males [27]. Modification of diet in renal disease (MDRD) equation used eGFR = 186 × (SCr (mg/dL))^−1.154^ × (age (years))^−0.203^ × (0.742 if female) × (1.21 if black) [28]. Cystatin C-based CKD-EPI equation used eGFR = 133 × min (cystatin C/0.8)^−0.499^ × 0.996^age^ (×0.932 if female) if cystatin C ≤ 0.8 and eGFR = 133 × (cystatin C/0.8)^−1.328^ × 0.996^age^ (×0.932 if female) if cystatin C > 0.8 [27]. Receiver operating characteristic (ROC) analyses were performed to assess ability of renal biomarkers (serum creatinine and serum cystatin C) and eGFR values to detect micro and macroalbuminuria (grade 2 and 3 DN).

### 2.5. Statistical Analysis

The data collected from the study were entered in MS Excel and analysed using SPSS version 20.0 software. Descriptive statistics (mean with standard deviation and median with interquartile ranges) were used to express demographic, anthropometric, and biochemical parameters. Student's *t*-tests and one-way ANOVA (for normally distributed variables) and the Mann–Whitney *U* test and Kruskal–Wallis test (nonnormally distributed) followed by post hoc analysis were performed for testing statistical significance. Pearson correlation analysis and Spearman's rho correlation analysis were performed for finding the correlation between normally distributed and nonnormally distributed variables, respectively. A chi-square test was performed to find the association of categorical variables. ROC curve analysis was performed to assess the diagnostic accuracy of various renal markers, and the area under curve (AUC) was calculated. Sensitivity and specificity for serum cystatin C and other markers were estimated by the cutoff value from ROC analysis.

## 3. Results

**Figure 1:**
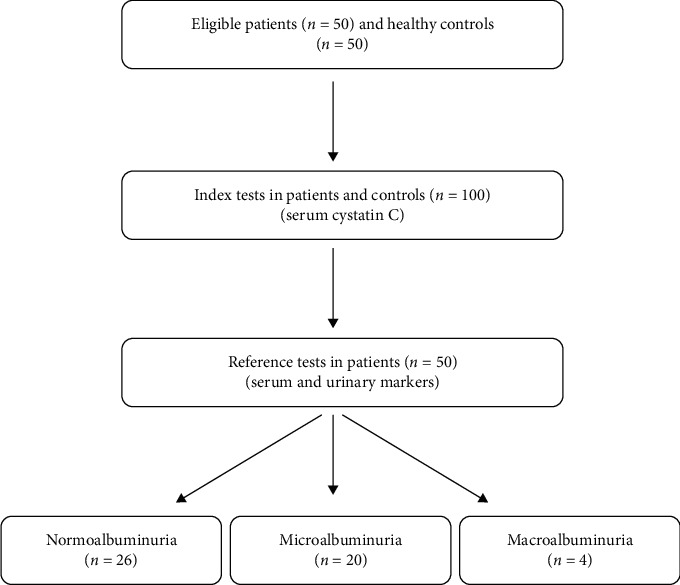
Study flow diagram.

Figure 1 describes the study flow. Among 50 cases of T2DM, 27 were male and 23 were female. In the control group, 34 were male and 16 were female. The general characteristics of T2DM patients are given in Table 1. Most of the cases were overweight/obese (56%) and on oral antidiabetic medication (70%). The average age ± SD of the cases and controls were 52.0 ± 11.0 and 40.8 ± 7.1 years, respectively (*p* < 0.001). The mean ± SD serum cystatin C was significantly higher in T2DM as compared to control (1.07 ± 0.38 and 0.86 ± 0.12 mg/dl, respectively, *p* < 0.001).

The mean ± SD bodyweight, BMI, W : H ratio, pulse, SBP, and DBP were 66.4 ± 12.6 kg, 26.2 ± 5.6 kg/m^2^, 1.03 ± 0.09, 78 ± 7, 125 ± 16 mm of Hg, and 77 ± 9 mm of Hg, respectively. The median duration of T2DM was five years. The serum biochemical data in the patients are given in Table 2. Based on the urinary albumin creatinine ratio (UACR) values, patients were divided into three groups: normoalbuminuria, microalbuminuria, and macroalbuminuria. A significant difference in HDL cholesterol (*p*=0.018) and serum cystatin C (*p* < 0.001) was observed among them. HDL cholesterol was significantly higher in patients with macroalbuminuria as compared to microalbuminuria (*p*=0.048) and normoalbuminuria (*p*=0.006). Similarly, patients with macroalbuminuria had higher cystatin C as compared to patients with microalbuminuria (*p* < 0.001) and normoalbuminuria (*p* < 0.001). Cystatin C was higher in microalbuminuric than normoalbuminuric patients (*p*=0.01).

Correlation of cystatin C with other anthropometric and biochemical parameters is given in Table 3. Cystatin C had a significant positive correlation with age (*r* = 0.323, *p*=0.022), duration of T2DM (*r* = 0.326, *p*=0.021), and UACR (*r* = 0.528, *p* < 0.001) and a significant negative correlation with eGFR CKD-EPI cystatin C (*r* = −0.925, *p* < 0.001).

The area under ROC curve for serum cystatin C (0.611, 95% CI: 0.450–0.772) was greater than for serum creatinine (0.429, 95% CI: 0.265–0.593) though nonsignificant. The area under ROC curve for eGFR CKD-EPI (cystatin C) (0.615, 95% CI: 0.450–0.780) was nonsignificantly higher than for eGFR CG (creatinine) (0.441, 95% CI: 0.280–0.602), eGFR MDRD (creatinine) (0.472, 95% CI: 0.307–0.637), and eGFR CKD-EPI (creatinine) (0.430, 95% CI: 0.264–0.596).

The sensitivity, specificity, positive predictive value (PPV), and negative predictive value (NPV) for serum creatinine, serum cystatin C, eGFR CG (creatinine), eGFR MDRD (creatinine), eGFR CKD-EPI (creatinine), and eGFR CKD-EPI (cystatin C) are given in Table 4. Serum cystatin C (cutoff set at 0.93 mg/L) had higher sensitivity (70.8% versus 66.7%) and specificity (61.5% versus 23.1%) than serum creatinine (cutoff set at 0.765 mg/dL). Similarly, higher sensitivity (75.0%) was seen with cystatin C-based eGFR CKD-EPI equation, but specificity was decreased when the cutoff point was set at 90 ml/min/1.73 m^2^.

## 4. Discussion

We measured serum cystatin C, serum creatinine, and urinary albumin creatinine ratio and calculated several eGFR values to assess DN in Nepalese patients suffering from T2DM. The mean age of patients was 52 years, with the median duration of diabetes five years. Around 56% of the patients were overweight or obese, and the majority of the patients (60%) had higher HbA1c (HbA1c > 7), which suggests an uncontrolled blood sugar level. Uncontrolled blood sugar is a leading cause for the development of diabetic complications, including nephropathy, and prompt management of hyperglycaemia can delay long-term complications [2]. Consistent with our previous study in Nepalese T2DM patients, most patients were overweight or obese and in their early fifties [29].

Diabetic nephropathy is characterized by albuminuria or decreased GFR, and urinary albumin creatinine ratio is a standard method to classify the different grades of DN [4–6]. Based on this ratio, frank proteinuria (macroalbuminuria: ≥300 mg/g of creatinine) was seen in 8% of T2DM patients. Microalbuminuria and normoalbuminuria were seen in 40% and 56% diabetic patients, respectively. Our findings are in line with the previous studies in Nepal. In one of those studies, 14.7% had frank proteinuria and 44.6% had microalbuminuria [8]. The other study reported frank proteinuria and microalbuminuria in 12.3% and 36.8% T2DM patients, respectively [30]. We observed higher HDL cholesterol in patients with macroalbuminuria as compared to microalbuminuria (*p*=0.048) and normoalbuminuria (*p*=0.006). This is in contrast with previous findings that macroalbuminuric patients have lower HDL cholesterol, which increases the risk for cardiovascular diseases [31]. Our result may be due to a small number of macroalbuminuric patients.

We observed a higher serum cystatin C level (1.07 ± 0.38 mg/dl versus 0.86 ± 0.12 mg/dl) in T2DM patients as compared to healthy controls. This is consistent with the cystatin C level seen in a previous study that tested the diagnostic efficiency of cystatin C in diagnosing renal impairment in preeclampsia patients (1.15 ± 0.37 mg/dl in patients versus 0.55 ± 0.1 mg/dl in control) [25]. Cystatin C is a molecule synthesized at a stable rate by all nucleated cells, and its concentration is not influenced by sex, protein ingestion, inflammation, or muscle mass [9, 10, 12]. In our study, serum cystatin C increased with UACR and decreased with eGFRCKD-EPI (Cyst C). Thus, patients with macroalbuminuria had higher cystatin C than patients with microalbuminuria (*p* < 0.001) and normoalbuminuria (*p* < 0.001). Cystatin C was also higher in microalbuminuric than normoalbuminuric patients (*p*=0.01). Similar to our findings, Jeon et al. reported a rise in serum cystatin C during progression from normoalbuminuria to macroalbuminuria, thereby revealing a positive correlation between serum cystatin C and UACR [15]. In addition, serum cystatin C was positively correlated with age and duration of diabetes, as seen in previous studies [32, 33]. Interestingly, while serum cystatin C increased during the progression of DN, no similar increment was seen for serum creatinine. Though serum creatinine is considered a specific marker, it is not very sensitive as its levels do not significantly increase until the GFR is reduced to less than 50% of normal [10]. Thus, our data support the applicability of cystatin C as an early marker of albuminuria in Nepalese patients. In the context of limited information regarding serum cystatin C in Nepalese T2DM patients, our findings provide evidence on the benefits of this blood marker.

In the present study, cystatin C had higher AUC, sensitivity, specificity, PPV, and NPV than serum creatinine. The AUC, sensitivity, specificity, PPV, and NPV values for cystatin C and creatinine are lower than observed in previous studies [15, 20, 32, 34]. It may be due to small sample size and a low degree of renal impairment in our study. Furthermore, eGFR equations based on serum creatinine had lower sensitivity and specificity as well as PPV and NPV values over cystatin C-based eGFR equations. The AUC of eGFR CKD-EPI (Cyst C) was nonsignificantly higher than that of eGFR CG (Scr), eGFR MDRD (Scr), and eGFR CKD-EPI (Scr). Several previous studies have compared the diagnostic value of creatinine-derived eGFR formula and cystatin C-derived formula [24, 27, 35, 36]. Those findings suggest better sensitivity of cystatin C-derived formula or a formula combining both cystatin C and creatinine than creatinine-derived equations to detect renal impairment at an early stage. Our current findings add country and ethnic-specific information about the benefit of cystatin C-based eGFR values to the existing literature.

Diabetic kidney disease usually takes around ten years to develop after diabetes; however, it may be present at the time of T2DM diagnosis [4]. Thus, screening for DN should be started at the time of diagnosis of DM. Early renal function assessment can help to prevent the development of ESRD and reduce the economic burden associated with hospital admission for dialysis or kidney transplant [4, 5, 11]. In this context of presence or progression of DN, the early rise of serum cystatin C and cystatin C-derived GFR could act as a better diagnostic tool than creatinine and creatinine-based GFR. Therefore, we emphasize the use of this marker routinely for tracking progression of nephropathy in Nepalese T2DM patients. This would also support in the early prediction of the risk of cardiovascular morbidity and mortality in T2DM patients.

This study has several limitations. This includes a small sample size, cross-sectional nature, and nonprobability sampling. This limits the diagnostic role for the progressive diabetic nephropathy disease, which requires the prospective study. A more reliable gold standard method of GFR measurement and 24-hour urinary albumin excretion rate (UAER) was not assessed.

## 5. Conclusion

Serum cystatin C concentration was significantly higher in T2DM patients than in healthy control. Concentration of cystatin C increases with the progression of nephropathy in T2DM patients. In addition, the cystatin C level rises with the longer period of diabetes and UACR. Thus, serum cystatin C had higher accuracy in diagnosing nephropathy than serum creatinine in the Nepalese T2DM patients.

## Figures and Tables

**Table 1:** General characteristics in the T2DM cases.

Characteristics	Categories	Frequency	Percentage (%)
Sex	M/F	34/16	68/38
Occupation	Housewife	18	36
	Service	11	22
	Agriculture	9	18
	Not specific	12	24
Medication	Oral	45	90
	Insulin only	3	6
	Insulin and oral	1	2
	None	1	2
Family history of DM	Yes/no	7/43	14
Vegetarian	Yes/no	4/46	80
Alcohol drinking past 1 year	Yes/no	10/40	20
Smoking	Yes/no	4/46	80
BMI categories	Underweight	2	4
	Normal weight	20	40
	Overweight	18	36
	Obese	10	20
Hypertension	Yes/no	15/25	30
Diabetic complications	Yes/no	7/43	14

The data are expressed as frequency and percentage of total cases.

**Table 2:** Variables according to different grades of diabetic nephropathy.

Variables	Total cases (*n* = 50)	Normoalbuminuria (*n* = 26)	Microalbuminuria (*n* = 20)	Macroalbuminuria (*n* = 4)	*P* value
Age (years)	51.6 ± 11.0	51 ± 10	51 ± 10	58 ± 22	0.536
Bodyweight (kg)	66.4 ± 12.6	66 ± 12.6	68.1 ± 12.8	60.3 ± 14.3	0.528
BMI (kg/m^2^)	26.2 ± 5.6	26.2 ± 6.2	26.6 ± 5.3	23.9 ± 2.7	0.686
W : H ratio	1.03 ± 0.09	1.04 ± 0.07	1.03 ± 0.11	1.00 ± 0.07	0.774
Pulse	78 ± 7	79 ± 6	77 ± 9	76 ± 10	0.581
SBP (mm of Hg)	125 ± 16	122 ± 13	126 ± 16	135 ± 31	0.311
DBP (mm of Hg)	77 ± 9	76 ± 9	79 ± 9	75 ± 13	0.556
Duration of DM (years)	5 (2, 10)	4 (1, 8)	6 (3, 13)	5 (1, 15)	0.275
Fasting blood glucose (mg/dl)	158.5 (137.0, 214.5)	157.5 (136.5, 214.0)	158.5 (143.7, 216.0)	169 (121.3, 486.8)	0.994
Postprandial (mg/dl)	281.5 (214.8, 382.3)	272.0 (214.5, 371.8)	295.5 (223.7, 371.3)	320.5 (175.8, 802.8)	0.886
HbA1c (%)	7.5 ± 2.0	7.3 ± 1.8	8.1 ± 2.4	7.1 ± 1.2	0.384
Triglycerides (mg/dl)	204.6 ± 96.9	184.8 ± 87.4	229.9 ± 107.6	206.9 ± 94.0	0.301
Total cholesterol (mg/dl)	197.1 ± 50.1	193.2 ± 52.7	206.0 ± 50.0	173.5 ± 27.4	0.454
HDL cholesterol (mg/dl)	40.4 ± 5.9	38.8 ± 6.2	41.1 ± 4.0	47.3 ± 7.7	0.018
Serum creatinine (mg/dl)	0.85 ± 0.23	0.87 ± 0.21	0.82 ± 0.22	0.88 ± 0.3	0.773
Serum cystatin C (mg/L)	1.07 ± 0.38	0.94 ± 0.22	1.07 ± 0.41	1.63 ± 0.66	<0.001
UACR	27.6 (10.6, 125.55)	11.9 (7.4, 21.0)	92.5 (56.3, 164.4)	618.4 (402.0, 705.2)	<0.001
eGFR_CG (creat)_	94.9 ± 36.2	90.9 ± 24.1	100.6 ± 39.5	92.1 ± 79.1	0.738
eGFR_MDRD (creat)_	96.5 ± 35.6	94.0 ± 30.1	99.3 ± 40.1	98.6 ± 55.0	0.706
eGFR_CKD-EPI (creat)_	109.3 ± 20.6	107.1 ± 17.4	112.7 ± 21.7	106.3 ± 36	0.634
eGFR_CKD-EPI (Cyst C)_	80.5 ± 26.3	94.1 ± 16.7	74.3 ± 27.5	45.7 ± 22.5	0.162

One-way ANOVA (for normally distributed variables) and the Kruskal–Wallis test (nonnormally distributed) were performed followed by post hoc analysis.

**Table 3:** Correlation of serum cystatin C with other parameters in T2DM cases.

Variables	Correlation coefficient	*P* value
Age (years)	0.323	0.022
Weight (kg)	−0.013	0.929
BMI (kg/m^2^)	−0.034	0.814
W : H ratio	0.211	0.141
Pulse	−0.059	0.682
SBP (mm of Hg)	0.261	0.067
DBP (mm of Hg)	0.162	0.261
Duration of DM (years)	0.326	0.021
Fasting blood glucose (mg/dl)	0.064	0.660
Postprandial (mg/dl)	0.087	0.547
HbA1c (%)	0.013	0.928
Triglycerides (mg/dl)	−0.020	0.892
Total cholesterol (mg/dl)	0.080	0.579
HDL cholesterol (mg/dl)	0.124	0.390
Serum creatinine (mg/dl)	0.211	0.141
UACR	0.528	<0.001
eGFRCG (creatinine)	−0.166	0.249
eGFRMDRD (creatinine)	−0.135	0.349
eGFRCKD-EPI (creatinine)	−0.251	0.078
eGFRCKD-EPI (cystatin C)	−0.925	<0.001

Pearson and Spearman correlation analysis was performed.

**Table 4:** ROC curve analysis of serum creatinine and cystatin C for diagnostic accuracy.

Parameters	Cutoff point	Sensitivity (%)	Specificity (%)	PPV (%)	NPV (%)
Serum creatinine	0.765 mg/dL	66.67	23.08	44.44	42.86
Serum cystatin C	0.93 mg/L	70.83	61.54	62.96	69.57
eGFRCG (creatinine)	90 ml/min/1.73 m^2^	75	46.15	56.25	66.67
eGFRMDRD (creatinine)	90 ml/min/1.73 m^2^	58.33	46.15	50	54.54
eGFRCKD-EPI (creatinine)	90 ml/min/1.73 m^2^	16.67	73.08	36.36	48.72
eGFRCKD-EPI (cystatin C)	90 ml/min/1.73 m^2^	75.0	46.15	56.25	66.67

## Data Availability

The data used to support the findings of this study are available from the corresponding author upon request.

## Abbreviations

ADA: American Diabetes Association
AUC: Area under curve
BMI: Body mass index
BP: Blood pressure
CKD: Chronic kidney disease
CKD-EPI: Chronic Kidney Disease Epidemiology Collaboration
DBP: Diastolic blood pressure
DM: Diabetes mellitus
DN: Diabetic nephropathy
eGFR: Estimated glomerular filtration rate
GFR: Glomerular filtration rate
ESRD: End-stage renal disease
HDL: High-density lipoprotein
MDRD: Modification of Diet in Renal Disease
ROC: Receiver operating characteristic
SBP: Systolic blood pressure
SCr: Serum creatinine
T2DM: Type 2 diabetes mellitus
UAE: Urinary albumin excretion
UACR: Urinary albumin creatinine ratio.

## References

[1] Zheng Y., Ley S. H., Hu F. B. (2018). Global aetiology and epidemiology of type 2 diabetes mellitus and its complications. *Nature Reviews Endocrinology*.

[2] Stolar M. (2010). Glycemic control and complications in type 2 diabetes mellitus. *The American Journal of Medicine*.

[3] Cade W. T. (2008). Diabetes-related microvascular and macrovascular diseases in the physical therapy setting. *Physical Therapy*.

[4] American Diabetes Association (ADA) (2017). Standard of medical care in diabetes–2017. *Diabetes Care*.

[5] Gross J. L., de Azevedo M. J., Silveiro S. P., Canani L. H., Caramori M. L., Zelmanovitz T. (2005). Diabetic nephropathy: diagnosis, prevention, and treatment. *Diabetes Care*.

[6] Papadopoulou-Marketou N., Kanaka-Gantenbein C., Marketos N., Chrousos G. P., Papassotiriou I. (2017). Biomarkers of diabetic nephropathy: a 2017 update. *Critical Reviews in Clinical Laboratory Sciences*.

[7] Maskey R., Shakya D., Sharma S., Karki P., Lavaju P. (2012). Diabetes mellitus related complications in out-patient clinic of tertiary care hospital. *Journal of College of Medical Sciences-Nepal*.

[8] Jha P., Das B. K., Shrestha S. (2010). Glycemic status, lipid profile and proteinuria in diabetic nephropathy. *JNMA Journal of Nepal Medical Association*.

[9] Campion C. G., Sanchez-Ferras O., Batchu S. N. (2017). Potential role of serum and urinary biomarkers in diagnosis and prognosis of diabetic nephropathy. *Canadian Journal of Kidney Health and Disease*.

[10] Currie G., McKay G., Delles C. (2014). Biomarkers in diabetic nephropathy: present and future. *World Journal of Diabetes*.

[11] Nichols G. A., Vupputuri S., Lau H. (2011). Medical care costs associated with progression of diabetic nephropathy. *Diabetes Care*.

[12] Fiseha T. (2015). Urinary biomarkers for early diabetic nephropathy in type 2 diabetic patients. *Biomarker Research*.

[13] Al-Rubeaan K., Siddiqui K., Al-Ghonaim M. A., Youssef A. M., Al-Sharqawi A. H., AlNaqeb D. (2017). Assessment of the diagnostic value of different biomarkers in relation to various stages of diabetic nephropathy in type 2 diabetic patients. *Scientific Reports*.

[14] Jeon Y. L., Kim M. H., Lee W. I., Kang S. Y. (2013). Cystatin C as an early marker of diabetic nephropathy in patients with type 2 diabetes. *Clinical Laboratory*.

[15] Jeon Y. K., Kim M. R., Huh J. E. (2011). Cystatin C as an early biomarker of nephropathy in patients with type 2 diabetes. *Journal of Korean Medical Science*.

[16] Aksun S., Özmen D., Özmen B. (2004). *β*2-microglobulin and cystatin C in type 2 diabetes: assessment of diabetic nephropathy. *Experimental and Clinical Endocrinology & Diabetes*.

[17] Gupta K., Nayyar S. B., Sachdeva J., Kumar P. (2017). Cystatin C in the early diagnosis of diabetic nephropathy and its correlation with albuminuria. *International Journal of Advances in Medicine*.

[18] Kim S. S., Song S. H., Kim I. J. (2013). Urinary cystatin C and tubular proteinuria predict progression of diabetic nephropathy. *Diabetes Care*.

[19] Takir M., Unal A. D., Kostek O., Bayraktar N., Demirag N. G. (2016). Cystatin-C and TGF-*β* levels in patients with diabetic nephropathy. *Nefrología (English Edition)*.

[20] Christensson A. G., Grubb A. O., Nilsson J.-A., Norrgren K., Sterner G., Sundkvist G. (2004). Serum cystatin C advantageous compared with serum creatinine in the detection of mild but not severe diabetic nephropathy. *Journal of Internal Medicine*.

[21] Groesbeck D., Köttgen A., Parekh R. (2008). Age, gender, and race effects on cystatin C levels in US adolescents. *Clinical Journal of the American Society of Nephrology*.

[22] Kramer H., Palmas W., Kestenbaum B. (2008). Chronic kidney disease prevalence estimates among racial/ethnic groups: the multi-ethnic study of atherosclerosis. *Clinical Journal of the American Society of Nephrology*.

[23] Teo B. W., Xu H., Wang D. (2012). Estimating glomerular filtration rates by use of both cystatin C and standardized serum creatinine avoids ethnicity coefficients in Asian patients with chronic kidney disease. *Clinical Chemistry*.

[24] Teo B. W., Zhang L., Guh J.-Y. (2018). Glomerular filtration rates in asians. *Advances in Chronic Kidney Disease*.

[25] Niraula A., Lamsal M., Baral N. (2017). Cystatin-C as a marker for renal impairment in preeclampsia. *Journal of Biomarkers*.

[26] Cockcroft D. W., Gault H. (1976). Prediction of creatinine clearance from serum creatinine. *Nephron*.

[27] Inker L. A., Schmid C. H., Tighiouart H. (2012). Estimating glomerular filtration rate from serum creatinine and cystatin C. *New England Journal of Medicine*.

[28] Stevens L. A., Coresh J., Feldman H. I. (2007). Evaluation of the modification of diet in renal disease study equation in a large diverse population. *Journal of the American Society of Nephrology*.

[29] Khatiwada S., Kc R., Sah S. K. (2015). Thyroid dysfunction and associated risk factors among Nepalese diabetes mellitus patients. *International Journal of Endocrinology*.

[30] Maharjan B. R., Bhandary S., Risal P., Sedhain A., Shakya P. R., Gautam M. (2010). Microalbuminuria and macroalbuminuria in type 2 diabetes. *Journal of Nepal Health Research Council*.

[31] Chen S.-C., Tseng C.-H. (2013). Dyslipidemia, kidney disease, and cardiovascular disease in diabetic patients. *The Review of Diabetic Studies*.

[32] Elbarbary H. S., El-Kafrawy N. A., Shoaib A. A., Kamal El-deen S. M. (2014). Serum cystatin C an early indicator of renal function decline in type 2 diabetes. *American Journal of Bio Science*.

[33] Hu Y., Liu F., Shen J. (2014). Association between serum cystatin C and diabetic peripheral neuropathy: a cross-sectional study of a Chinese type 2 diabetic population. *European Journal of Endocrinology*.

[34] Mussap M., Vestra M. D., Fioretto P. (2002). Cystatin C is a more sensitive marker than creatinine for the estimation of GFR in type 2 diabetic patients. *Kidney International*.

[35] Avinash S., Singh V. P., Agarwal A. K., Chatterjee S., Araya V. (2015). Identification and stratification of diabetic kidney disease using serum cystatin C and serum creatinine based estimating equations in type 2 diabetes: a comparative analysis. *JAPI: Journal of the Association of Physicians of India*.

[36] Domingueti C. P., Fóscolo R. B., Simões e Silva A. C. (2016). Evaluation of creatinine-based and cystatin C-based equations for estimation of glomerular filtration rate in type 1 diabetic patients. *Archives of Endocrinology and Metabolism*.

